# Caution in studying and interpreting the lupus metabolome

**DOI:** 10.1186/s13075-020-02264-2

**Published:** 2020-07-17

**Authors:** Ting Zhang, Chandra Mohan

**Affiliations:** Department of biomedical engineering, University of Houston, Houston, TX 77204 USA

**Keywords:** Lupus, Metabolome, Pathways, Steroid-dependent

## Abstract

Several metabolomics studies have shed substantial light on the pathophysiological pathways underlying multiple diseases including systemic lupus erythematosus (SLE). This review takes stock of our current understanding of this field. We compare, collate, and investigate the metabolites in SLE patients and healthy volunteers, as gleaned from published metabolomics studies on SLE. In the surveyed primary reports, serum or plasma samples from SLE patients and healthy controls were assayed using mass spectrometry or nuclear magnetic resonance spectroscopy, and metabolites differentiating SLE from controls were identified. Collectively, the circulating metabolome in SLE is characterized by reduced energy substrates from glycolysis, Krebs cycle, fatty acid β oxidation, and glucogenic and ketogenic amino acid metabolism; enhanced activity of the urea cycle; decreased long-chain fatty acids; increased medium-chain and free fatty acids; and augmented peroxidation and inflammation. However, these findings should be interpreted with caution because several of the same metabolic pathways are also significantly influenced by the medications commonly used in SLE patients, common co-morbidities, and other factors including smoking and diet. In particular, whereas the metabolic alterations relating to inflammation, oxidative stress, lipid peroxidation, and glutathione generation do not appear to be steroid-dependent, the other metabolic changes may in part be influenced by steroids. To conclude, metabolomics studies of SLE and other rheumatic diseases ought to factor in the potential contributions of confounders such as medications, co-morbidities, smoking, and diet.

## Introduction

Systemic lupus erythematosus (SLE) is a chronic autoimmune disease characterized by multi-system involvement with activation of multiple immune cell types and the production of diverse autoantibodies. The pathogenesis of SLE is not fully understood, but genetic and environmental factors both contribute to the development and progression of the disease [1]. Genomic, transcriptomic, and proteomic changes have been extensively studied in lupus. The metabolome is a relatively novel dimension which has received increasing attention in all fields including autoimmunity. On the one hand, individual metabolites have been ascribed roles in modulating immune cells and shaping immune responses, and these studies in SLE have been providing novel insights on this disease [2–4]. On the other hand, global metabolomics have been conducted on a more comprehensive scale [5–11], shedding light on all categories of metabolites. In carefully reviewing these studies, interesting lessons are emerging, as detailed below.

## Circulating metabolome in SLE

We searched PubMed using “lupus” and “metabolomic or metabolome or metabolic” as keywords in English literatures published up till May 2020. This yielded 172 publications. All these publications were reviewed, and only studies using serum or plasma samples in the metabolomics studies were included, narrowing down qualified studies to 13 [5–17]. One study was excluded as half of the SLE patients in that study had concomitant infections [16], and another was excluded since it exclusively focused on pregnant SLE patients [17]. Eleven studies investigating the circulating SLE metabolome are reviewed in this communication, as listed in Table 1 [5–15]. In these studies, serum or plasma samples from SLE patients and controls have been assayed using liquid chromatography mass spectrometry (LC/MS), gas chromatography mass spectrometry (GC/MS), or ^1^H nuclear magnetic resonance spectroscopy (NMR). The number of study subjects ranged from 18 to 80, with the number of differential metabolites between SLE and healthy controls identified per study ranging from 4 to 319. The metabolites altered in SLE mostly fell into several major metabolic pathways, including carbohydrate metabolism, amino acid metabolism, lipid metabolism, and inflammation-related pathways (Table 2).

**Table 1:** Eleven studies investigating the serum or plasma metabolome in SLE patients

Study	Country	Differential Metabolites*	Biofluid	Platform	Patients	Controls	Corrections	Confounding factors mentioned
Ouyang 2011 [5]	China	27	Serum	^1^H-NMR	64 SLE	30 RA, 35 HC	Age, sex, race	M, S
Wu 2012 [16]	USA	319	Serum	LC/MS, GC/MS	20 SLE	9 HC	Age, sex, race, BMI	M, Co-M
Bengtsson 2016 [7]	Sweden	20	Serum	GC/MS	30 SLE	18 HC, 19 SSc, 20 pSS	Age, sex, race	M
Guleria 2016 [8]	India	19	Serum	^1^H-NMR	22 SLE, 40 LN	30 HC	Age, sex, race	M
Yan 2016 [9]	China	41	Serum	GC/MS	80 SLE	57 HC	Age, sex, race, BMI	M
Li 2017 [12]	China	23	Serum	LC/MS	32 LN	30 INS, 28 HC	Age, sex, race, BMI	None
Shin 2017 [10]	Korea	13	Plasma	GC/MS	41 SLE	41 HC	Age, sex, race	M
Guleria 2018 [11]	India	17	Serum	^1^H-NMR	18 LN	18 LNT, 30 HC	Age, sex, race	M
Li 2019 [14]	China	50	Serum	LC/MS	17 SLE	17 HC	Age, sex, race	None
Bellocchi 2019 [13]	Italy	4	Plasma	LC/MS	27 SLE	23 pSS, 11 PAPS, 26 UCTD, 27 HC	Age, sex, race	M
Zhang 2020 [15]	China	55	Serum	LC/MS	32 SLE	25 HC	Age, sex, race, BMI	M

*Co-M* co-morbidities, *GC/MS* gas chromatography mass spectrometry, *HC* healthy control, *INS* idiopathic nephrotic syndrome, *LC/MS* liquid chromatography mass spectrometry, *LN* lupus nephritis, *LNT* lupus nephritis after treatment, *M* medications, *NMR*^1^H nuclear magnetic resonance spectroscopy, *PAPS* primary anti-phospholipid syndrome, *pSS* primary Sjögren’s syndrome, *RA* rheumatoid arthritis, *S* smoking, *SLE* systemic lupus erythematosus, *SSc* systemic sclerosis, *UCTD* undifferentiated connective tissue disease

*Differentially expressed metabolites between SLE (or LN if all patients were LN) and HC

**Table 2:**
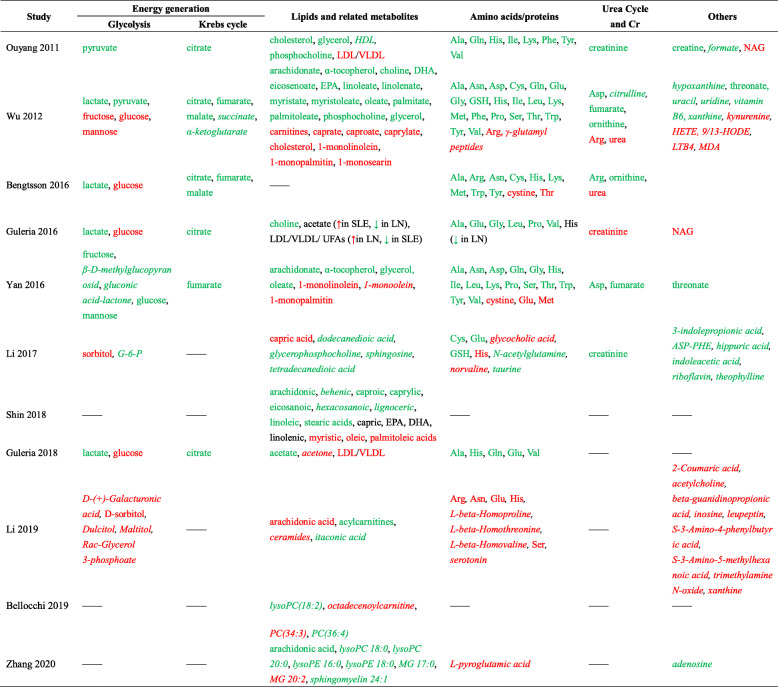
Altered serum/plasma metabolites in SLE patients, based on eleven published studies

Metabolites which discriminate SLE from controls are listed in this table. Assayed metabolites which did not distinguish SLE from controls are listed only if they were evaluated in more than one study. Green font indicates downregulation and red font indicates upregulation of serum/plasma metabolites in SLE compared to controls, while metabolites in black font remained unchanged or changes were inconsistent in subgroups of SLE patients. Metabolites in italics were those that were only measured in one study

*Ala* alanine, *Arg* arginine, *Asn* asparagine, *Asp* aspartic acid, *ASP-PHE*l-aspartyl-l-phenylalanine, *Cys* cysteine, *DHA* docosahexaenoic acid, *EPA* eicosapentaenoic acid, *G-6-P* glucose 6-phosphate, *Gln* glutamine, *Glu* glutamic acid, *Gly* glycine, *GSH* glutathione, *HDL* high-density lipoprotein, *HETE* hydroxyeicosatetraenoic acid, *His* histidine, *HODE* hydroxyoctadecadienoic acid, *Ile* isoleucine, *LDL* low-density lipoprotein, *Leu* leucine, *LN* lupus nephritis, *LTB4* leukotriene B4, *Lys* lysine, *LysoPC* lysophosphatidylcholine, *LysoPE* lysophosphatidylethanolamine, *MDA* malonaldehyde, *Met* methionine, *MG* monoacylglycerol, *NAG* N-acetyl glycoproteins, *PC* phosphatidylcholine, *Phe* phenylalanine, *Pro* proline, *Ser* serine, *SLE* systemic lupus erythematosus, *Thr* threonine, *Trp* tryptophan, *Tyr* tyrosine, *UFA* unsaturated fatty acids, *Val* valine, *VLDL* very low-density lipoprotein

**Fig. 1:**
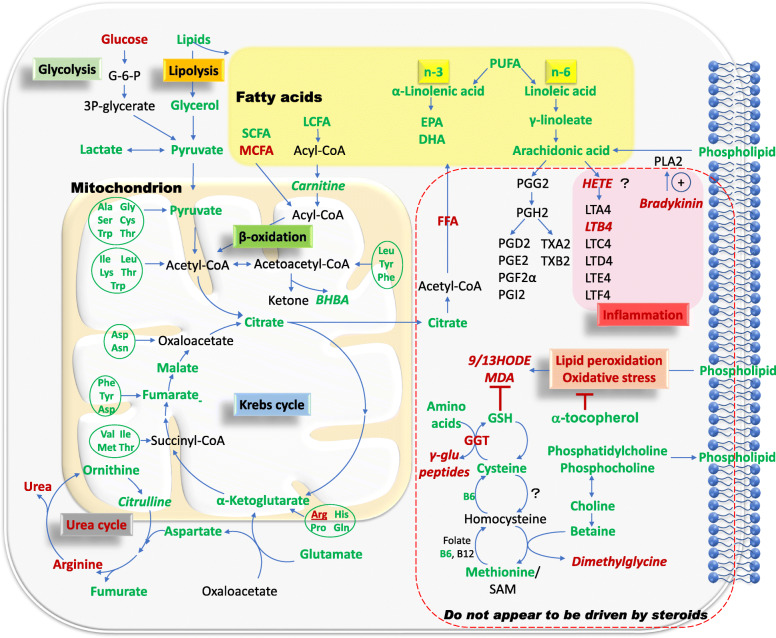
An overview of the major pathways implicated in serum/plasma metabolomics alteration in SLE. Metabolites elevated in SLE are shown in red font, while reduced metabolites are in green font. Metabolites in italics were only measured in one study. Pathways that appear unlikely to be steroid dependent include the elevation of bradykinin/leukotrienes and lipid peroxidation, as shown boxed with a red dashed line. Pathways outside this box may potentially be the consequence of steroids, based on the known metabolic effects of steroids [18–20]. *Ala* alanine, *Arg* arginine, *Asn* asparagine, *Asp* aspartic acid, *BHBA* 3-hydroxybutyrate, *Cys* cysteine, *DHA* docosahexaenoic acid, *EPA* eicosapentaenoic acid, *FFA* free fatty acids, *GGT* gamma-glutamyltransferase, *Gln* glutamine, *Glu* glutamic acid, Gly glycine, *GSH* glutathione, *HETE* hydroxyeicosatetraenoic acid, *His* histidine, *HODE* hydroxyoctadecadienoic acid, *Ile* isoleucine, *LCFA* long-chain fatty acids, *Leu* leucine, *LT* leukotriene, *Lys* lysine, *MCFA* medium-chain fatty acids, *MDA* malonaldehyde, *Met* methionine, *PG* prostaglandin, *Phe* phenylalanine, *Pro* proline, *PUFA* polyunsaturated fatty acid, *SAM* S-adenosyl-methionine, *SCFA* short-chain fatty acids, *Ser* serine, *Thr* threonine, *Trp* tryptophan, *TX* thromboxane, *Tyr* tyrosine, *Val* valine

Overall, metabolites required for energy generation were decreased in serum or plasma in patients with SLE across multiple studies. The pathways implicated in this respect include glycolysis, Krebs cycle, fatty acid β oxidation, and glucogenic and ketogenic amino acid metabolism (Fig. 1). Glucose is initially metabolized into pyruvate in the cytosol, which is then converted into lactate under anaerobic conditions or is transported into the mitochondria where it participates in the Krebs cycle under aerobic conditions. The Krebs cycle, or the tricarboxylic acid cycle (TCA cycle), is the final catabolic pathway which oxidizes carbohydrates, fatty acids as well as amino acids that enter the cycle, and accounts for the generation of 90% of the energy released from food [21]. In multiple studies, glycolysis appeared to be subdued in SLE, as indicated by elevated glucose but reduced pyruvate and lactate. Likewise, Krebs cycle intermediates were reduced in SLE in multiple studies (Fig. 1, Table 2), alluding to reduced activity of the Krebs cycle in SLE [5–11]. Whether the apparent sluggishness of energy metabolism pathways contributes to the generalized fatigue documented in SLE patients remains unknown, as discussed elsewhere [6].

Amino acids display wide-ranging metabolic and regulatory roles, including intracellular protein turnover, gene expression, synthesis and secretion of hormones, nutrient metabolism, oxidative defense, and immune function [22]. During amino acid catabolism, the carbon skeleton and the amino groups are channeled into separate but interconnected pathways, namely the Krebs cycle and urea cycle, respectively. Amino acids entering the Krebs cycle may contribute to energy generation, but in humans, the oxidative energy derived from the catabolism of amino acids comprises only a minor fraction [23]. Most of the amino acids assayed in SLE, including both gluconeogenic and ketogenic amino acids, were generally downregulated in the peripheral blood (Fig. 1, Table 2). The catabolic product of the amino group is ammonia, which is converted to urea via the urea cycle and then excreted in the urine. Metabolites in the urea cycle were measured in two of the eleven metabolomics studies [6, 7]. Arginine, the immediate precursor metabolite of urea, was increased in one study while decreased in the other. However, urea was increased in both studies, suggesting increased activity of the urea cycle in SLE [6, 7].

**Fig. 2:**
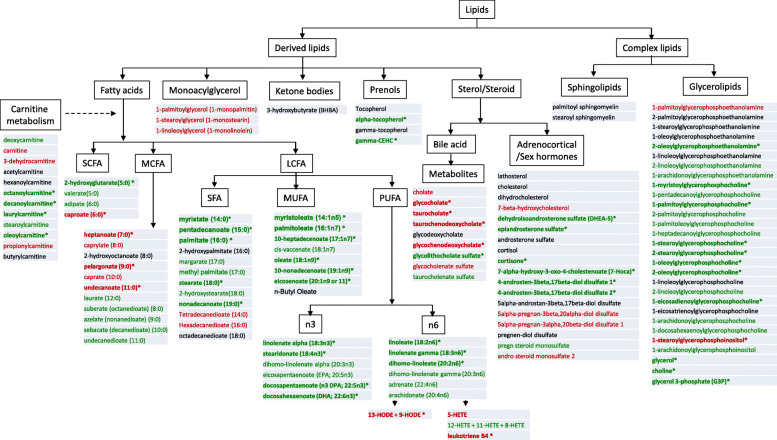
An overview of the lipid alterations in SLE sera. This data was drawn from the study that interrogated the largest number of lipid metabolites in SLE [6]. Green: decreased in all patients or severe lupus. Red: increased in all patients or severe lupus. Black: no change in all patients or severe lupus. Bolded with asterisks: significantly changed in either mild or severe lupus (*p* < 0.05). Of the lipids listed in this figure, arachidonate, caproate, caprylate, eicosanoate, linolenate, stearate, EPA, DHA, linoleneate, myristate, oleate, and palmitoleate were also altered in the study by Shin et al. [10], as detailed in Table 2. *LCFA* long-chain fatty acids, *MCFA* medium-chain fatty acids, *MUFA* mono-unsaturated fatty acids, *PUFA* polyunsaturated fatty acid, *SCFA* short-chain fatty acids, *SFA* saturated fatty acids

Lipids are fundamental nutrients with crucial and diverse functions, ranging from storage of energy to being the major structural elements of biological membranes. Other lipids act as enzyme cofactors, electron carriers, hormones, and intracellular messengers [24]. Lipid metabolism has been reported to be extensively altered in SLE patients, with possible roles in modulating immune responses and disease progression [25, 26]. Multiple changes in lipid profiles have been documented in the completed metabolomics studies. The most comprehensive screen of circulating lipids in SLE was reported by Wu et al. [6], with the other published metabolomics studies reporting only a subset of these changes, owing mostly to differences in the platform used (Table 1). Employing the LC-MS platform, Wu et al. interrogated a significantly larger number of lipid species than the other studies [6], and the lipid changes observed in SLE are portrayed in Fig. 2. Generally, long-chain fatty acids (LCFA), including the n3 and n6 polyunsaturated fatty acids (PUFA), were significantly reduced in the serum of SLE patients, but medium-chain fatty acids (MCFA) and free fatty acids (FFA) were increased [6], as indicated in Fig. 1, Fig. 2, and Table 2. To enter the mitochondrial matrix for further β oxidation, short-chain fatty acids (SCFA) can directly pass across the inner mitochondrial membrane, while LCFA needs transportation assistance from carnitines [21], which were mostly decreased in SLE patients when compared with healthy controls [6].

With respect to membrane lipids, most phosphocholines were reduced in two independent studies, possibly reflecting increased cell turnover. In one study, this reduction also extended to phosphatidylethanolamine (Fig. 2) [5]. Evidence of augmented oxidative stress was noted in SLE patients, as indicated by increased lipid peroxidation products including malonaldehyde (MDA), 9-hydroxyoctadecadienoic acid (HODE), and 13-HODE, and decreased antioxidants including α-tocopherol, glutathione, and its precursors [6, 27]. Lipid peroxidation, in association with free radical activity and cellular damage of membranes and possibly other organelles and/or DNA [21], has been associated with arterial and renal manifestations in SLE [28].

As with carbohydrates and amino acids, the alteration of lipid metabolites in SLE was mostly consistent across the different metabolomics studies. For instance, arachidonic acid, an n6 PUFA and a precursor for many inflammatory mediators including leukotrienes, thromboxanes, and prostaglandins, was mostly decreased in SLE patients in several studies [6, 9, 10, 15]. However, some disparities were also noted. For example, capric, caproic, and caprylic acids were increased, while eicosenoic, myristic, oleic, and palmitoleic acids were decreased in SLE patients in one study [6], but opposite results were noted in a different study and cohort [10]. Similarly, linolenic acid together with docosahexaenoic acid (DHA) and eicosapentaenoic acid (EPA) was either decreased or remained unchanged in SLE [6, 10].

## Potential impact of concomitant medications on metabolites in SLE

When interpreting the results of metabolomics studies, it is important to keep in mind that metabolites are extensively influenced by various coexisting factors other than the disease per se. One of the most significant categories is medications used in SLE, including glucocorticoids (GCs) and multiple immunosuppressants.

GCs are powerful steroid hormones with anti-inflammatory and immunosuppressive properties, and patients with SLE will almost inevitably have to use GCs for disease management. The commonly used GCs are prednisone, methylprednisolone, and occasionally dexamethasone (DEX). Of relevance to this discussion, GCs result in various adverse side effects including metabolic disturbances affecting glucose, lipids, and proteins. In rats treated with DEX 2.5 mg/kg twice a week for 14 weeks, serum metabolites showed reduced phenylalanine, lysine, and arginine, with increased tyrosine, hydroxyproline, and acylcarnitines, with impacts on gluconeogenesis, protein catabolism, and adipose degradation [18]. It has also been documented in healthy volunteers that a single 4-mg dose of DEX triggered significant dysregulation of up to 150 metabolites in plasma. Following the administration of DEX, glucose, lactate, mannose, and several amino acids were elevated. Most individual lipids including phosphatidylcholines and triacylglycerols, saturated, and mono-unsaturated fatty acids (MUFA) and PUFAs including linoleic acid, arachidonic acid, α-linolenic acid, EPA, and DHA were all decreased, while very long-chain (C22/C24) fatty acids remained either unchanged or only slightly increased. Acylcarnitines were upregulated. The abovementioned alternation in lipid profiles is consistent with increased lipolysis with low to no impact on peroxisomal oxidation [19]. Notably, several of these metabolic alterations parallel the findings reported in SLE. In another study, when patients with Cushing’s syndrome or adrenocortical adenomas with or without hypercortisolism were compared with hormonally normal controls, patients with hypercortisolism showed lower levels of short and medium-chain acylcarnitines as well as branched-chain and aromatic amino acids, but higher polyamines levels [20]. Taken together, it is plausible that a subset of the metabolomics alterations reported in SLE may in part be attributed to GCs while others may be less impacted by these drugs, as indicated in Fig. 1.

While the influence of GCs on glucose, lipids, and amino acids is well understood, the direct impact of GCs on metabolites implicated in lipid peroxidation as well as bradykinins/leukotrienes has not been systemically investigated. However, it has been reported that there was no significant association between GC usage and serum metabolites related to oxidative stress, glutathione generation, and selected inflammation-related pathways, including MDA, glutathione, leukotriene B4 (LTB4), and gamma-glutamyltransferase 1 (GGT1) in individual datasets [6]. These findings are consistent with the published metabolic effects of GC in mice and humans [18, 19]. Based on these observations, we have demarcated in Fig. 1 a subset of the observed metabolic changes in SLE that may be relatively independent of steroids, whereas other metabolic alterations observed in SLE may largely be the consequence of steroids. Clearly, further studies are warranted to tease out the metabolic alterations in SLE that are directly driven by GCs. Given the potential influence of GCs on plasma metabolites, dosages and treatment durations should always be adjusted for in interpreting the results from metabolomics studies of SLE or other rheumatic diseases.

Hydroxychloroquine (HCQ) is another medication commonly used in patients with SLE, and this drug has been recognized as having favorable effects on glucose and lipid metabolism. Growing evidence has confirmed its beneficial impact on cardiovascular risk, diabetes, and dyslipidemia [29]. HCQ was associated with serum low-density lipoprotein (LDL) level reduction in patients with SLE [30], and also with decreased triglycerides and very low-density lipoprotein (VLDL), as well as increased total high-density lipoprotein (HDL) [31]. For several other immunosuppressants used in SLE, glucose and lipid metabolic dysregulation is a well-recognized complication. Cyclosporine and tacrolimus have been associated with increased serum levels of cholesterol, triglycerides, and LDL in a dose-dependent manner, as a result of enhanced lipolysis, reduced lipid storage, and expression of lipogenic genes in the adipose tissue [32]. Both cyclosporine and tacrolimus are associated with hyperglycemia and hyperlipidemia [33, 34]. Azathioprine inhibits purine synthesis and DNA replication, but there is no evidence that it disrupts glucose or lipid metabolism [33]. Similarly, no influence of cyclophosphamide on metabolites has been reported.

Because of the overlapping influence of concomitant medications on the serum metabolome in SLE, it is essential to carefully evaluate the potential influence of medications before conclusions are drawn. In 9 out of the 11 studies reviewed, concomitant medications were either generally mentioned or explicitly listed, but omitted in the remaining two studies (Table 1). Metabolomics studies in drug-naïve new-onset SLE patients before the initiation of treatment will be critical to capture a more precise picture of the metabolic alterations in SLE. In this respect, the metabolic changes in murine lupus may be particularly informative.

## Influence of other confounding factors on metabolites in SLE

Smoking has a strong impact on serum metabolites. When metabolic profiles were compared between current smokers and never smokers, twenty-one serum metabolites were significantly different between the two groups, mainly consisting of lipids and amino acids [35]. Besides, it has also been reported that in asthma patients, several arachidonic acid metabolites were increased in smokers when compared with never smokers [36]. Only one out of the eleven SLE metabolomics studies reviewed here clearly indicated that they had excluded smokers when recruiting patients, while others did not mention the smoking status of patients. Given the potential overlap between the smoking-associated metabolome and the apparent SLE-associated metabolome, it becomes important to pay attention to this confounding factor in future metabolomics studies.

Co-morbidities including hypertension, diabetes mellitus, dyslipidemia, cardiovascular disease, and infections also have a significant impact on serum metabolites. It has been reported that several carbohydrate metabolites, amino acids, choline-containing phospholipids, and acylcarnitines are associated with type 2 diabetes (T2D) or the risk of T2D [37–39]. When T2D was accompanied by complications including hypertension and coronary heart disease, differentially expressed serum metabolites included amino acids, lipids, carbohydrate metabolism, and Krebs cycle metabolites [40]. Similarly, in patients with hypertension and heart failure, diagnostic or prognostic values have been established for several metabolites, including those overlapping with the SLE metabolome [41, 42]. Unfortunately, co-morbidities were listed in only a few studies in SLE (Table 1), and this clearly warrants more careful investigation.

SLE primarily affects women of childbearing age, and pregnancy is known to increase the risk of disease flares [43]. Serum metabolites in pregnant SLE patients have been reported to be different from that of healthy pregnant women and may predict adverse pregnancy outcomes in SLE [17]. However, the specific contributions of the disease (SLE), the pregnant state, and concurrent mediations to the observed metabolomics landscape in these patients remains a black box.

## Gut microbiota, diet, and serum metabolites in SLE

Gut microbiota closely interacts with the host immune system and has become a growing field of interest in SLE [44]. *Enterococcus gallinarum* plays a causative role in a mouse model of SLE [45], and dysbiosis of the gut microbiome in SLE patients correlates with clinical manifestations and disease activity [46–48]. Serum antibodies against *Ruminococcus gnavus* correlated directly with SLEDAI and antinative DNA levels, inversely with C3 and C4, and were highest in patients with active nephritis [48]. Interventions in the gut microbiota affect lupus severity, progression, and treatment in lupus mice [49, 50].

Gut microbiota is also tightly associated with host metabolism, including circulating serum metabolites [51–56]. In patients with SLE, intestinal dysbiosis is associated with altered fecal SCFA and serum FFA [52]. In the eleven studies reviewed, one study measured both serum metabolomics and gut microbiome profiles in patients with systemic autoimmune diseases, demonstrating a strong association between intestinal microbiota and certain serum metabolites. Serum acylcarnitines were positively correlated with a Prevotella-enriched cluster, and both acylcarnitines and phospholipids were negatively correlated with butyrate-producing bacteria [13]. Along the same lines, recombinant microbes have been reported to improve glucose and lipid metabolism in diet-induced obese rodents [57], while genetic manipulation of *Clostridium sporogenes* altered the serum levels of metabolites such as pyruvate, lactate, and acetate in gnotobiotic mice [53].

Daily food intake cannot only directly shape the circulating metabolome, it can indirectly contribute by altering gut microbiota ecology [58–62]. Thus, for example, dietary vitamin D, vitamin A, and PUFAs in SLE have been shown to modulate the composition and function of gut microbiota, which in turn can impact innate and adaptive immunity [46]. Although it is clear that the daily diet and the gut microbiome can both have a major impact on the circulating metabolome, these may be the most difficult confounding factors to correct for in metabolomics studies.

## Serum metabolites versus intracellular metabolites in SLE

It is also noteworthy that while circulating metabolites are easily accessed and measured, the more relevant metabolic pathways are those within the pathogenic cells and end-organs. Perl et al. interrogated the metabolome of peripheral blood lymphocytes (PBL) in SLE patients, where the pentose phosphate pathway was most prominently impacted. Cysteine was depleted, while cystine and kynurenine were among the most increased metabolites [63]. These alterations in intracellular cysteine, cystine, and kynurenine in SLE PBL are consistent with the observations in circulation [6, 7, 9].

As discussed earlier, arachidonic acid is generally decreased in the serum of SLE patients. Similarly, there was a decrease of arachidonic acid in peripheral blood monocytes, but this was not observed in T lymphocytes in SLE patients [64]. Any apparent discrepancies observed between the serum metabolome and selected intracellular metabolomes in SLE may partly be explained by the fact that the serum metabolome is likely to be reflective of metabolic activity in all cells and tissues in the body, including endothelial cells, the liver, adipose tissue, and the microbiome, besides circulating blood cells. The extent to which different cells in the body contribute to the serum metabolome is currently a black box. Future studies that examine the metabolomes of multiple tissues/cells together with paired serum metabolome from the same SLE patients are warranted.

## Conclusions and recommendations

Taking all studies together, the circulating SLE metabolome is suggestive of reduced activity in energy-generating pathways, including glycolysis, Krebs cycle, fatty acid β oxidation, and glucogenic and ketogenic amino acid metabolism; decreased LCFA, but elevated MCFA and FFA, accompanied by augmented peroxidation and inflammation; and enhanced activity of the urea cycle, possibly reflecting increased catabolic activity. While metabolic alterations relating to inflammation, oxidative stress, lipid peroxidation, and glutathione generation do not appear to be steroid-dependent, the other metabolic changes may in part be influenced by steroids or other confounding variables. Hence, future metabolomics studies should factor in the following recommendations.

- ►Caution should be exercised in interpreting metabolomics studies in SLE (and other rheumatic diseases) since the metabolome is greatly influenced by multiple confounders including diet, medications, lifestyle, and co-morbidities. Examination of drug-naïve SLE patients will provide valuable insights on the SLE specific metabolome.
- ►When planning future metabolomics studies, it would be important to correct not only for demographic variables, but also for the patients’ smoking status, co-morbidities, and medications.
- ► Since the use of differing platforms (e.g., GC/MS, LC/MS, NMR, etc.) capture different domains of metabolites, and vary extensively in their species diversity, it becomes important to standardize assay techniques, so that differences in technology is no longer a confounding factor.
- ► Multi-center studies that examine SLE patients from different continents may be useful in delineating SLE-specific metabolomics changes that are relatively independent of ethnicity, diet, and other environmental influences.
- ► Of all potential confounding factors, the diet (and the microbiome) may be the most intractable to address. For starters, the use of well-accepted standardized food intake surveys in future metabolomics studies may give a handle on this variable.
- ► The above recommendations apply not only to metabolomics studies in SLE, but also to similar investigations in other rheumatic diseases such as rheumatoid arthritis and primary Sjögren’s syndrome, where these same confounding factors have been consistently overlooked [65–72].

## Data Availability

Not applicable
